# Comparative Study of PBS and PBSA Biodegradable Nanofibers Produced by Electro-Blow Spinning for Sustainable Air Filtration Applications

**DOI:** 10.3390/mi17080929

**Published:** 2026-08-03

**Authors:** Eman Elnabawy, Nagham M. Elberishy, Ahmed Backar, Ali Toptas, Ali Kilic, Islam Shyha, Mohamed A. Daha

**Affiliations:** 1 School of Computing, Engineering and the Built Environment, Edinburgh Napier University, Edinburgh EH10 5DT, UK; i.shyha@napier.ac.uk; 2 Department of Industrial Engineering, College of Engineering, University of Business and Technology, Jeddah 21448, Saudi Arabia; n.elberishy@ubt.edu.sa; 3 Production Engineering Department, Faculty of Engineering, Alexandria University, Alexandria 21544, Egypt; ahmed.backar@alexu.edu.eg (A.B.); m.daha@alexu.edu.eg (M.A.D.); 4 TEMAG Labs, Faculty of Textile Tech and Design, Istanbul Technical University, Istanbul 34437, Türkiye; atoptas34@gmail.com (A.T.); alikilic@itu.edu.tr (A.K.)

**Keywords:** nanofibres, air filtration, electro-blow spinning, biodegradable polymers

## Abstract

Poly Butylene Succinate (PBS) and Poly Butylene Succinate-co-Adipate (PBSA) are biodegradable polyesters that have attracted considerable attention for nanofibre fabrication due to their environmentally friendly characteristics and favourable material properties. PBS nanofibres are known by relatively high rigidity, thermal stability and crystallinity, making them suitable for applications that require structural strength. However, the stiffness of PBS may reduce its flexibility and limit its processing performance in some applications. In contrast, PBSA nanofibres incorporate adipic acid into the polymer chain, which results in lower crystallinity, greater flexibility and reduced melting temperature. These characteristics enhance the elasticity and processability of PBSA-based materials. The differences in mechanical and thermal behaviour between PBS and PBSA arise from the modification of the polymer backbone in PBSA, making it more appropriate for applications that demand adaptable and flexible materials, such as tissue engineering and air filtration. Both nanofibre systems show significant potential for sustainable technologies. In this study, a comparative evaluation of PBS and PBSA nanofibres was conducted to investigate their morphology, crystallinity, mechanical behaviour and filtration performance. The results show that PBS nanofibres exhibit a higher crystallinity of approximately 45%. In contrast, PBSA nanofibres demonstrate a significantly higher strain, reaching nearly twice that of PBS nanofibres. Regarding filtration performance, both materials achieved filtration efficiencies of up to 93%, while maintaining relatively low pressure drops of 210 Pa for PBS and 200 Pa for PBSA. Furthermore, corona discharge treatment was applied to enhance electrostatic capture and maintain filtration efficiency during extended operation for up to seven days.

## 1. Introduction

Biodegradable polymers such as PLA, PBS, PBSA, PBAT and PHA are materials capable of naturally decomposing through the activity of microorganisms, including bacteria and fungi. During this process, the polymers are converted into environmentally benign products such as water, carbon dioxide and biomass. Unlike conventional petroleum-based plastics, many biodegradable polymers are produced from renewable feedstocks such as starch, cellulose or plant-derived sugars. Their ability to break down naturally helps mitigate environmental pollution and reduces the accumulation of plastic waste in landfills and marine environments [1,2,3].

**Figure 1:**
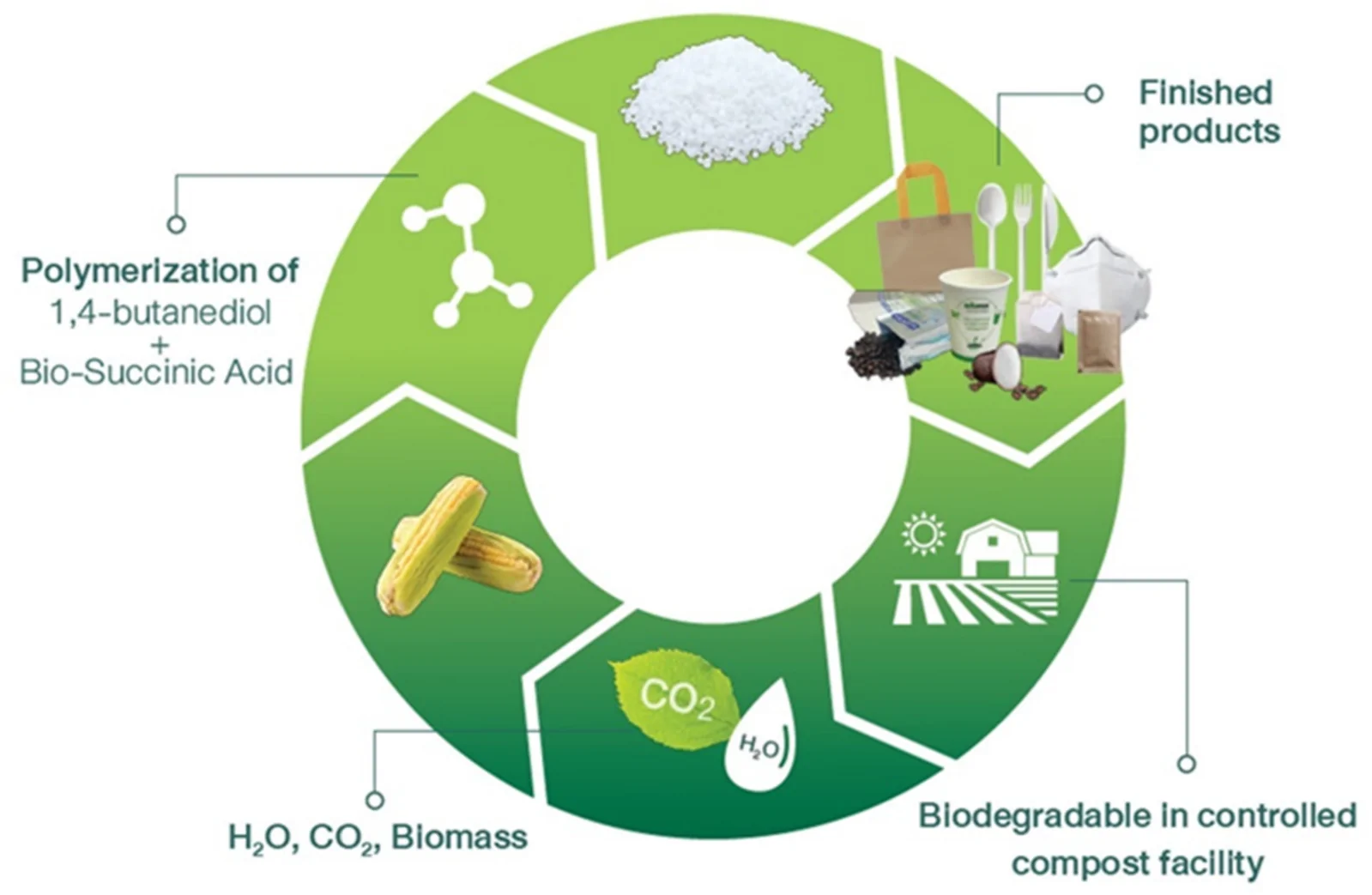
The life cycle of PBS from polymerisation to degradation [6].

PBS and poly PBSA are biodegradable aliphatic polyesters that have received growing attention due to their sustainability and favourable material properties [4]. These polymers can be produced from renewable resources and exhibit excellent biodegradability, which makes them attractive candidates for environmentally friendly materials in several industrial sectors. Among their various forms, nanofibres have emerged as particularly promising because of their high surface area, adjustable mechanical behaviour and potential for surface functionalisation. PBS and PBSA nanofibres also exhibit significant porosity and flexibility, characteristics that make them well suited for filtration and biomedical uses. In addition, these materials degrade into non-toxic products under suitable environmental conditions, supporting global efforts to reduce reliance on petroleum-based plastics and limit plastic pollution [5] (shown in Figure 1).

Although PBS and PBSA originate from similar monomer units and share biodegradable characteristics, they differ significantly in structure, mechanical performance and degradation behaviour. PBS generally exhibits higher rigidity and slower biodegradation, which makes it suitable for applications where mechanical strength and stability are required. PBSA, on the other hand, is more flexible and softer, and degrades more rapidly, making it appropriate for applications where flexibility and faster environmental breakdown are desirable [7]. Previous studies have also explored the fabrication of PBS nanofibres using electrospinning techniques. For example, Cooper et al. examined the effect of different solvent systems, including chloroform, dimethylformamide and dimethyl sulfoxide, on nanofibre formation. Their results showed that a chloroform/DMSO mixture produced fibres with fewer bead defects, while a polymer concentration of 15 wt.% yielded uniform fibres with relatively small diameters [8].

Structurally, PBS is a linear aliphatic polyester composed of repeating butylene succinate units, whereas PBSA is a copolymer in which a portion of the succinic acid units is replaced by adipic acid. This modification introduces greater flexibility into the polymer backbone. As a result, PBS typically shows a higher degree of crystallinity because of its more regular molecular structure, which contributes to its rigidity and higher melting temperature (114–120 °C). In contrast, the incorporation of adipic acid disrupts chain regularity in PBSA, reducing its crystallinity and lowering its melting temperature to approximately 70–90 °C. These structural differences also influence biodegradation behaviour. Although PBS is biodegradable, its higher crystallinity slows the degradation process because tightly packed polymer chains are less accessible to microbial or enzymatic attack. PBSA contains more amorphous regions and adipate segments that are more susceptible to hydrolysis, resulting in a faster degradation rate. This characteristic makes PBSA particularly suitable for environmentally degradable products such as compostable packaging and disposable filtration materials [9,10,11].

Recent studies have also explored the potential of biodegradable nanofibres based on PBS and PBSA for air filtration applications. Due to their high surface-area-to-volume ratio, interconnected porous structure and tuneable fibre diameter, these materials are capable of efficiently capturing fine airborne particles. Electrospun PBS nanofibres have been reported to exhibit high filtration efficiency against submicron particles while maintaining relatively low pressure drop, which is a key requirement for breathable filtration media. The relatively high crystallinity and mechanical stability of PBS contribute to the formation of uniform fibre networks that enhance particle interception and diffusion mechanisms. PBSA nanofibres, on the other hand, offer greater flexibility and elasticity because of their lower crystallinity and more amorphous structure. These characteristics allow PBSA-based membranes to form highly porous and adaptable filtration layers that can improve airflow and reduce resistance during filtration. In addition, biodegradable polyester nanofibres such as PBS and PBSA can be further enhanced through electrostatic charging or surface modification, which improves their ability to capture ultrafine particles through electrostatic attraction. As a result, these materials have attracted increasing interest as sustainable alternatives to conventional petroleum-based filtration media, particularly for applications such as disposable face masks, indoor air purification and environmental filtration systems.

Although the thermal and mechanical properties of PBS and PBSA have been extensively reported, comparative studies investigating how these polymers behave during electro-blow spinning and how their resulting nanofibrous structures influence air filtration performance are still limited. In this work, PBS and PBSA nanofibres were fabricated under identical electro-blow spinning conditions to establish direct relationships between polymer structure, fibre morphology, crystallinity, mechanical behaviour, wettability, corona charging and filtration performance. This comparative approach provides practical insights into the selection of biodegradable polyesters for sustainable air filtration applications and demonstrates the potential of electro-blow spinning as a scalable manufacturing route for biodegradable filter media.

## 2. Experimental Work

### 2.1. Materials

PBS and PBSA were supplied by BioPBS™ (Mitsubishi Chemical Europe GmbH, Düsseldorf, Germany). The PBS and PBSA grades used in this study were FZ91 and FD92, respectively. Chloroform (Sigma-Aldrich, Dorset, UK) was used as the solvent without further purification. The key physical, thermal and mechanical properties of PBS and PBSA pellets provided by the manufacturer are summarised in Table 1.

### 2.2. Nanofibre Manufacturing

**Figure 2:**
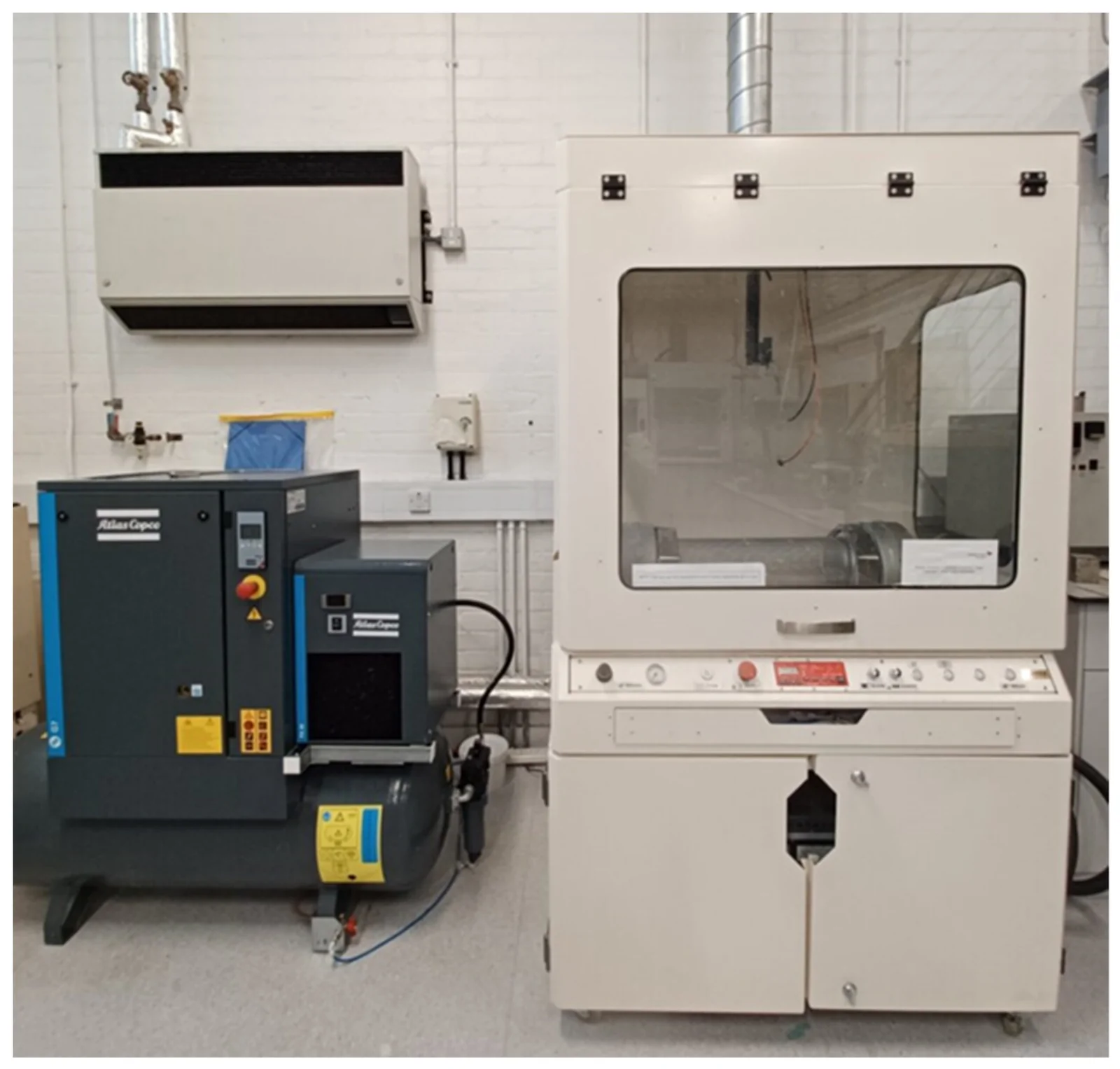
Image of the EBS setup used for nanofibre manufacturing.

PBS and PBSA nanofibres were produced using the electro-blow spinning (EBS) technique, which combines the principles of electrospinning and solution blowing in a single hybrid system. The EBS apparatus was provided by AREKA Advanced Technologies (Istanbul, Turkey). Polymer solutions containing 10 wt.% PBS or PBSA were prepared by dissolving the polymers in chloroform and stirring the mixtures overnight at room temperature to obtain homogeneous solutions. The prepared solution was then loaded into a 100 mL disposable syringe and mounted on the syringe pump of the EBS system. The EBS nozzle consisted of a concentric configuration with two separate channels. The inner channel delivered the polymer solution at a flow rate of 10 mL h^−1^, while compressed air supplied by an Atlas Copco air compressor was introduced through the outer channel. A high-voltage power supply (CZE1000R Spellman, Hauppauge, NY, USA) was applied to the nozzle at 20 kV to assist fibre formation. The resulting polymer jet was directed towards a rotating cylindrical collector positioned 45 cm from the nozzle (Figure 2). Nanofibres were deposited onto a polypropylene nonwoven substrate, which was later removed from the collector for subsequent characterisation.

### 2.3. Nanofibre Characterisations

#### 2.3.1. Morphological and Structural Analysis

The morphology and average fibre diameter of the PBS and PBSA nanofibres were examined using scanning electron microscopy (SEM). Samples were first cut into small sections, mounted onto carbon adhesive tabs and coated with a thin layer of platinum to improve conductivity. SEM imaging was performed using a TESCAN VEGA microscope (Brno, Czech Republic) operating at an accelerating voltage of 5 kV. Fibre diameter distributions were determined using ImageJ software (version 1.54t). Chemical structures and functional groups were analysed using Fourier-transform infrared spectroscopy (FTIR) (PerkinElmer Frontier, Shelton, CT, USA) in attenuated total reflectance (ATR) mode. Spectra were recorded within the range of 4000–400 cm^−1^ at a resolution of 5 cm^−1^. All spectra were normalised before comparison of peak intensities.

#### 2.3.2. Thermal Analysis

The thermal behaviour of the nanofibres was investigated using a PerkinElmer 8000 differential scanning calorimeter (CT, USA). Approximately 5–10 mg of each sample was sealed in stainless-steel pans. The samples were heated from 20 °C to 200 °C at a heating rate of 10 °C min^−1^ during both the first and second heating cycles. Subsequently, the samples were cooled back to 20 °C at the same rate and held for two minutes. The degree of crystallinity (χc) for each sample was calculated using the formula below:

(1) $$\chi c\;=\;(\Delta H_{m}/\Delta H_{m}^{0})\;\times \;100$$

where $\Delta H_{m}$ represents the melting enthalpy of the sample, and $\Delta H_{m}^{0}$ denotes the melting enthalpy assuming 100% crystalline PBS of 110 J/g and PBSA of 142 J/g [12,13,14].

#### 2.3.3. Crystallinity

The crystalline structure and phase composition of the nanofibres were analysed by powder X-ray diffraction (XRD) using an X’Pert3 diffractometer (Malvern Panalytical, Worcestershire, UK) equipped with Cu Kα radiation (λ = 1.54056 Å). Measurements were conducted at 45 kV and 40 mA over a 2θ range of 2–60°. The scanning step size was 0.0263°, and the total acquisition time for each measurement was approximately 10 min.

#### 2.3.4. Contact Angle and Pore Size Measurements

Surface wettability was assessed by static water contact angle measurements using a Krüss DSA100 (Hamburg, Germany) contact angle goniometer. The system was equipped with a high-resolution camera and drop-shape analysis software to determine the contact angle at the three-phase interface. A stainless-steel 29-gauge needle with an inner diameter of 0.184 mm was used to dispense droplets with a controlled volume while minimising vibration. A 2 μL droplet of distilled water was placed on the nanofibre surface, and measurements were taken immediately (t = 0 s). Four measurements were performed for each sample at room temperature. Pore size distribution of the nanofibre membranes was determined using capillary flow porometry (Anton Paar Porometer 3G, Boynton Beach, FL, USA). The nanofibre mats were saturated with a wetting liquid and subjected to increasing air pressure up to 3 bar. The bubble point (largest pore), mean pore size and smallest pore diameter were then calculated.

#### 2.3.5. Mechanical Analysis

The tensile behaviour of PBS and PBSA nanofibre mats was measured using a Textechno FAVIMAT+ single-fibre tester (Mönchengladbach, Germany). Rectangular specimens measuring 2 mm in length and 5 mm in width were prepared for testing. Measurements were performed at room temperature using a crosshead speed of 10 mm min^−1^, a gauge length of 2 mm and a load cell of 200 cN. Tensile stress was calculated by dividing the applied load by the initial cross-sectional area of the specimen, where the cross-sectional area was determined from the measured specimen width and thickness. The tensile strength, elongation at break and Young’s modulus were determined for five specimens of each sample, and the average values were reported.

#### 2.3.6. Air Filtration Measurement

**Figure 3:**
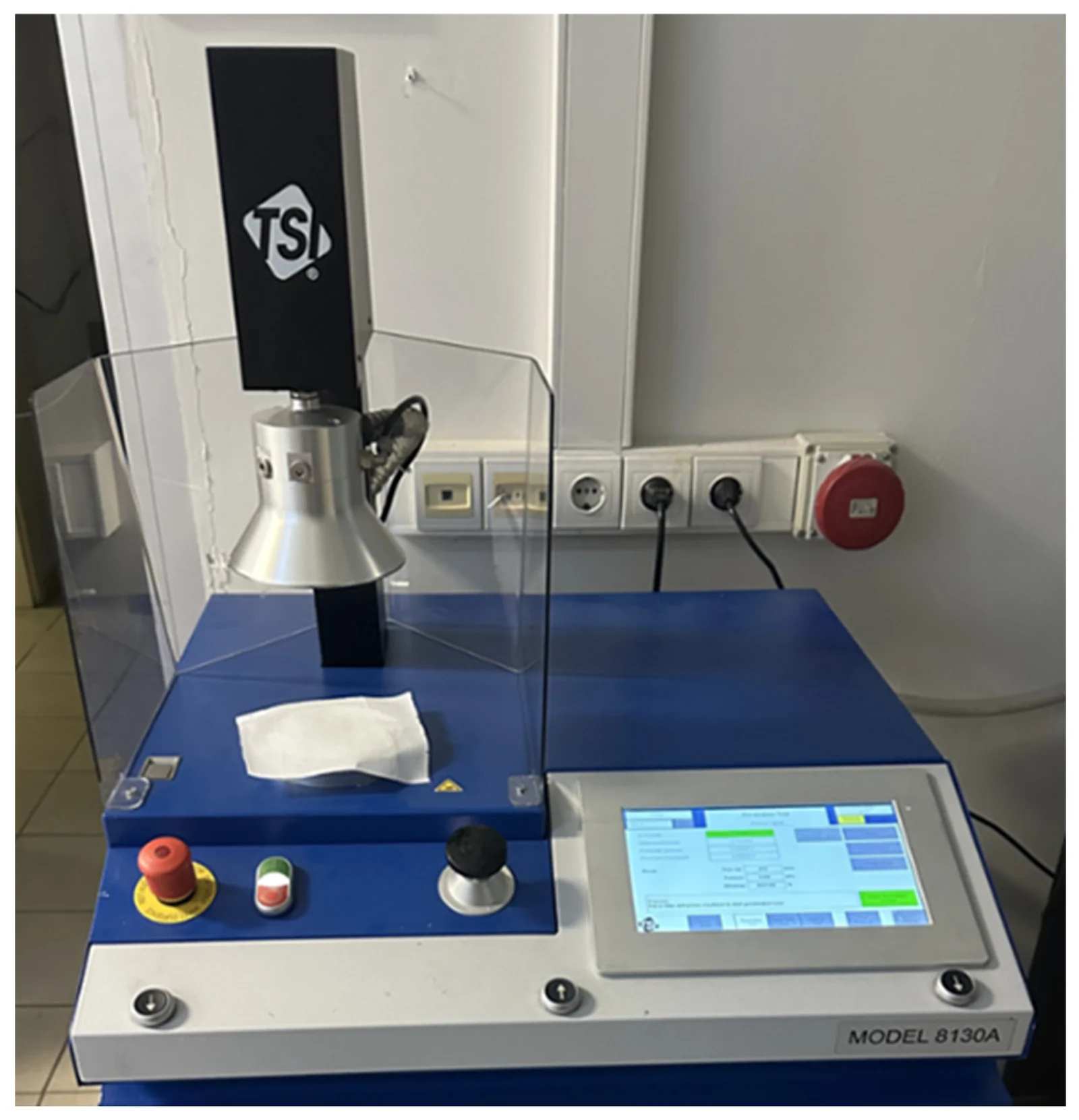
The automated TSI air filtration setup.

Filtration performance was evaluated using an automated filter tester (model 8130A, TSI Inc., Shoreview, MN, USA) to determine efficiency at the most penetrating particle size (MPPS), typically around 0.3 μm (Figure 3). Sodium chloride (NaCl) aerosols were generated from a 2 wt.% aqueous solution, producing solid particles with an average diameter of 0.26 ± 0.07 μm. Nanofibre membranes with an effective filtration area of 100 cm^2^ were exposed to the aerosol stream at a flow rate of 95 L min^−1^. All tests were conducted according to the European standard for respiratory protective devices (EN 149). Filtration efficiency (η) and quality factor (QF) were calculated using Equations (2) and (3). To enhance electrostatic particle capture, corona charging was applied using a Chargemaster 5 system (Simco-Ion, Hatfield, PA, USA). The nanofibre samples were placed 15 cm below the discharge electrode and exposed to a negative DC voltage of 20 kV for 3 min. After charging, the samples were immediately tested to evaluate the improvement in filtration efficiency. The stability of corona charging was evaluated indirectly through changes in filtration efficiency after storage rather than by direct measurements of surface potential or charge density.

(2) η=1−Cdown/Cup

(3) QF=−ln(1−η)/ΔP

Here, Cdown refers to the particle concentration downstream of the filter, Cup represents the upstream concentration, and ΔP denotes the pressure drop across the filter.

## 3. Results and Discussion

### 3.1. Morphological Properties

**Figure 4:**
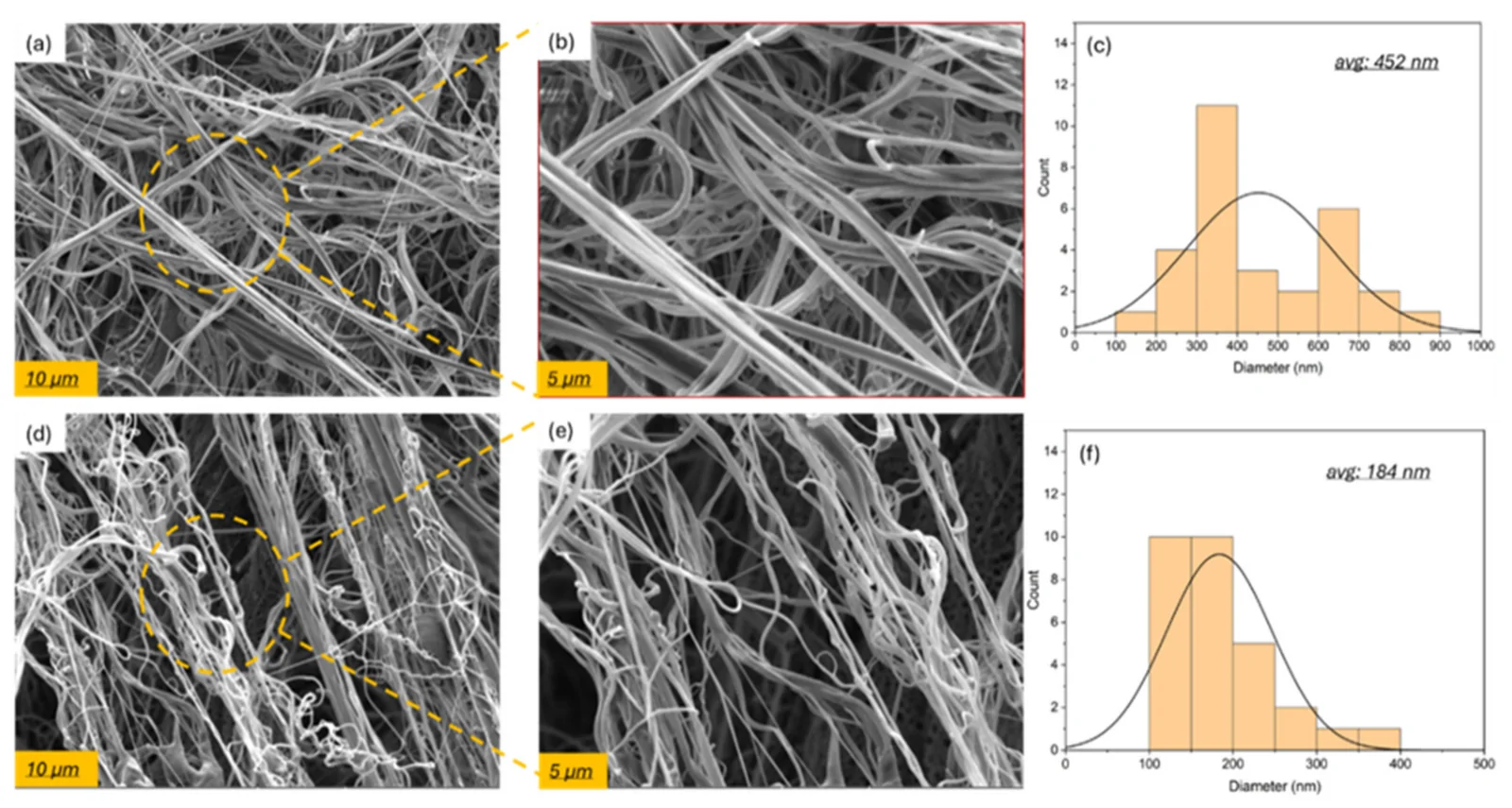
SEM images and fibre diameter distributions of the PBS and PBSA nanofibres: (**a**) low-magnification PBS; (**b**) high-magnification PBS; (**c**) PBS fibre diameter distribution; (**d**) low-magnification PBSA; (**e**) high-magnification PBSA; and (**f**) PBSA fibre diameter distribution.

The surface morphology of the PBS and PBSA nanofibres is illustrated in Figure 4 SEM images show that both nanofibre mats possess relatively uniform structures without visible bead defects, indicating that stable spinning conditions were achieved during the electro-blow spinning process [15]. PBS nanofibres exhibit a more uniform and homogeneous fibre distribution with well-defined individual fibres and an average diameter of 184 ± 32 nm. In comparison, PBSA nanofibres display a significantly larger average diameter of 452 ± 24 nm and tend to form partially bundled fibre structures. This behaviour results in a less uniform fibre network compared with PBS nanofibres.

The observed morphological differences can be attributed to the intrinsic properties of the polymers, particularly their crystallinity and intermolecular interactions during fibre formation [16]. The absence of bead defects and the formation of continuous fibres for both materials confirm that the electro-blow spinning parameters used in this study were suitable for producing high-quality nanofibre membranes with uniform distribution and minimal fibre aggregation.

### 3.2. Structural Properties

**Figure 5:**
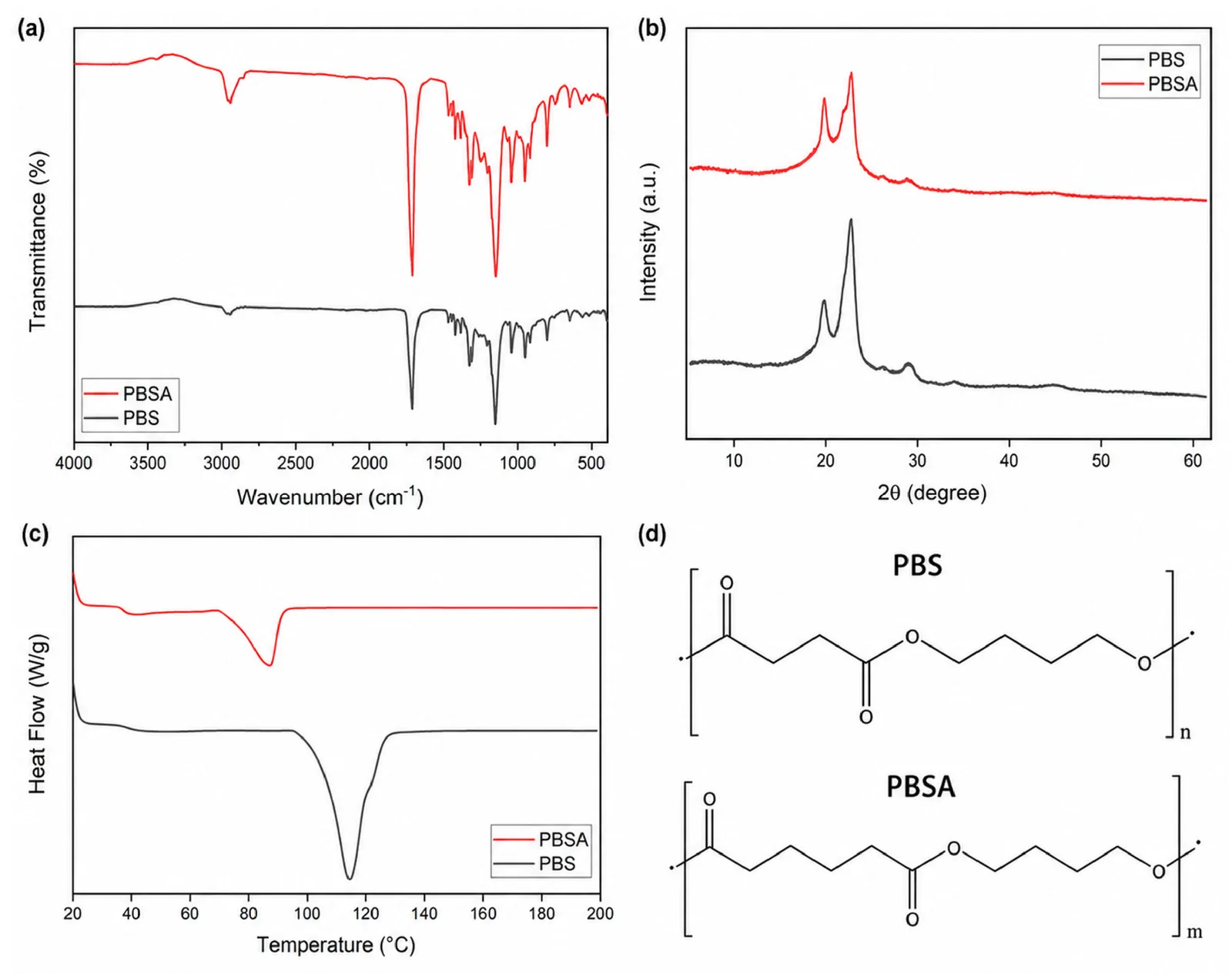
FTIR spectra (**a**), XRD spectra (**b**), DSC spectra (**c**), and the chemical structure formula of PBS and PBSA monomers (**d**).

The structural characteristics of PBS and PBSA nanofibres were analysed using FTIR, XRD and DSC techniques to investigate their chemical structure, crystallinity and thermal behaviour, as well as crystallinity. The FTIR spectra of PBS and PBSA nanofibres are presented in Figure 5a.

The FTIR spectra of PBS and PBSA nanofibres confirm the characteristic chemical structure of aliphatic polyesters. Both polymers exhibit strong absorption bands at approximately 1715 cm^−1^, corresponding to the ester carbonyl (C=O) stretching vibration, which is the characteristic functional group of PBS and PBSA. The bands located at 2945 and 2860 cm^−1^ are attributed to the asymmetric and symmetric stretching vibrations of methylene (CH_2_) groups in the butylene segments. Peaks observed at approximately 1460–1415 cm^−1^ arise from CH_2_ bending vibrations, while the absorption bands between 1260 and 1045 cm^−1^ correspond to asymmetric and symmetric C–O–C stretching vibrations of the ester linkage. Additional bands around 955, 805 and 720 cm^−1^ are associated with CH_2_ rocking and skeletal vibrations characteristic of PBS-based polyesters. Although both polymers exhibit similar functional groups, the absorption bands of PBSA are broader and slightly less defined than those of PBS, reflecting the lower crystallinity and greater molecular disorder introduced by the adipate units. These observations are consistent with the XRD and DSC results, which demonstrate a significantly lower crystallinity for PBSA than PBS [17,18].

The XRD patterns further highlight differences in crystallinity between the two nanofibre systems. PBS nanofibres exhibit sharp and intense diffraction peaks, indicating a highly ordered crystalline structure. In contrast, PBSA nanofibres display broader peaks with lower intensity, suggesting a lower degree of crystallinity and a more disordered molecular arrangement. The main diffraction peaks for PBS appear at 19.4°, 21.5° and 28.8°, corresponding to the crystal planes (020), (021) and (110), respectively [19,20]. As shown in Figure 5b, the intensity of these peaks is significantly lower for PBSA nanofibres, confirming their reduced crystalline content.

The thermal behaviour of the nanofibres was examined using DSC, and the results are presented in Figure 5c. PBS nanofibres show a melting temperature of approximately 120 °C, which is consistent with their higher crystallinity. In contrast, PBSA nanofibres exhibit a lower melting temperature of around 80 °C and a broader melting peak, indicating a higher proportion of amorphous regions. The crystallinity values calculated from the DSC curves reveal that PBS nanofibres possess a crystallinity of approximately 45%, whereas PBSA nanofibres show a significantly lower crystallinity of 21.5%.

### 3.3. Contact Angle and Pore Size Analysis

**Figure 6:**
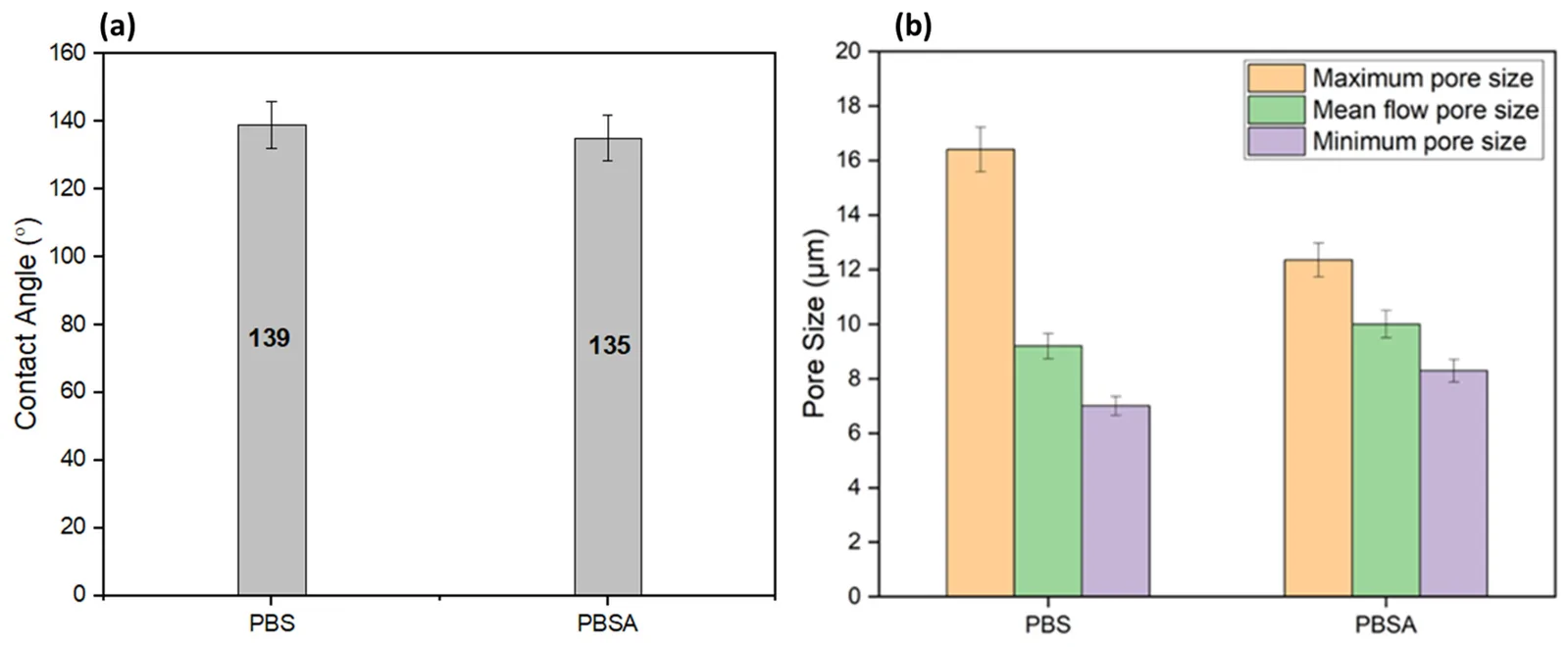
Contact angle (**a**) and pore size analysis (**b**) of the PBS and PBSA nanofibres.

The wettability and pore structure of PBS and PBSA nanofibre membranes were evaluated using contact angle and capillary flow porometry measurements. Both polymers exhibit hydrophobic behaviour, with water contact angles of 139° for PBS and 135° for PBSA, as shown in Figure 6a. The slightly higher contact angle observed for PBS indicates a marginally more hydrophobic surface compared with PBSA. In terms of pore structure, PBSA nanofibres show a slightly larger mean pore diameter of approximately 10 µm, whereas PBS nanofibres exhibit a smaller mean pore size of about 8.5 µm (Figure 6b). Similarly, the smallest pore sizes were measured as 8.2 µm for PBSA and 7.2 µm for PBS. The bubble point analysis revealed values of 12 µm for PBSA and 16 µm for PBS, indicating a more uniform pore size distribution in the PBSA nanofibre mat.

These variations in pore characteristics are closely related to the differences in fibre morphology and packing density. The larger fibre diameter and partially bundled structure of PBSA nanofibres lead to a more open network structure and consequently larger pores. In contrast, the finer fibres and more homogeneous distribution observed in PBS nanofibres result in a denser fibre network with smaller pore sizes. Such differences in surface wettability and pore structure are important factors that influence the suitability of nanofibre membranes for applications such as air filtration, tissue engineering and drug delivery, where surface interactions and porous architecture play critical roles [10,21,22].

### 3.4. Mechanical Properties

The tensile properties of PBS and PBSA nanofibres were investigated to evaluate their mechanical performance. Significant differences were observed between the two materials, mainly due to variations in crystallinity and fibre morphology. PBS nanofibres exhibit higher tensile strength and Young’s modulus, which can be attributed to their higher crystalline content and the formation of well-defined individual fibres. These characteristics contribute to greater stiffness and improved load-bearing capacity. However, this increased rigidity results in lower flexibility and reduced elongation at break.

**Figure 7:**
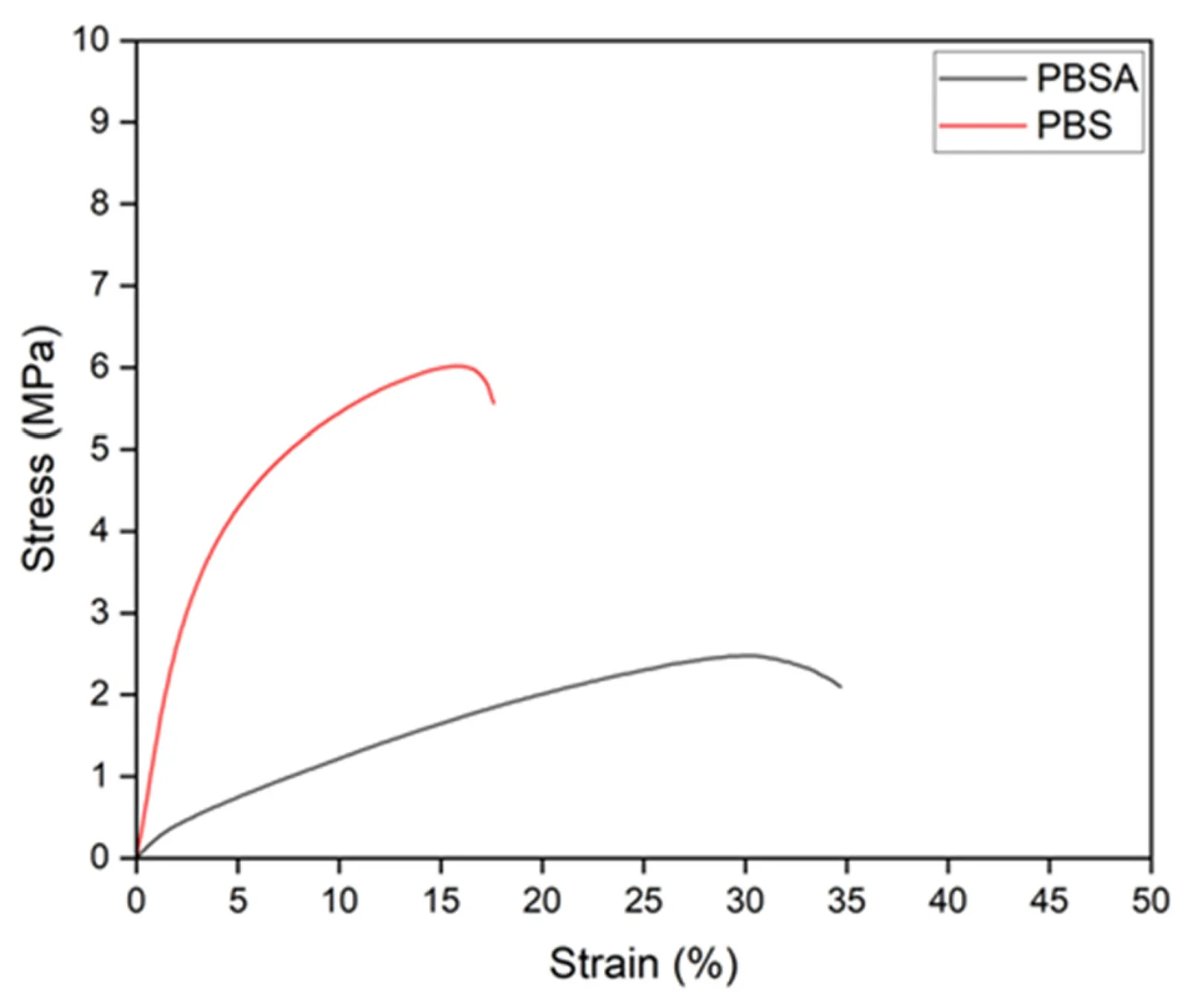
Stress−strain curves of PBS and PBSA nanofibres.

In contrast, PBSA nanofibres demonstrate lower tensile strength and modulus because of their lower crystallinity and bundled fibre structure. Nevertheless, PBSA nanofibres show significantly higher elongation at break, indicating superior flexibility and stretchability under mechanical stress [5,6,9]. The stress–strain curves presented in Figure 7 illustrate these differences clearly. PBS nanofibres exhibit a maximum stress of approximately 6 MPa, whereas PBSA nanofibres show a lower value of around 2 MPa. However, PBSA nanofibres demonstrate a much higher strain at break of 35%, compared with 17% for PBS nanofibres. Young’s modulus values further confirm these trends. PBS nanofibres show a modulus of approximately 150 MPa, reflecting their higher stiffness, while PBSA nanofibres exhibit a significantly lower modulus of about 15 MPa. The detailed mechanical properties are summarised in Table 2.

### 3.5. Air Filtration Performance

**Figure 8:**
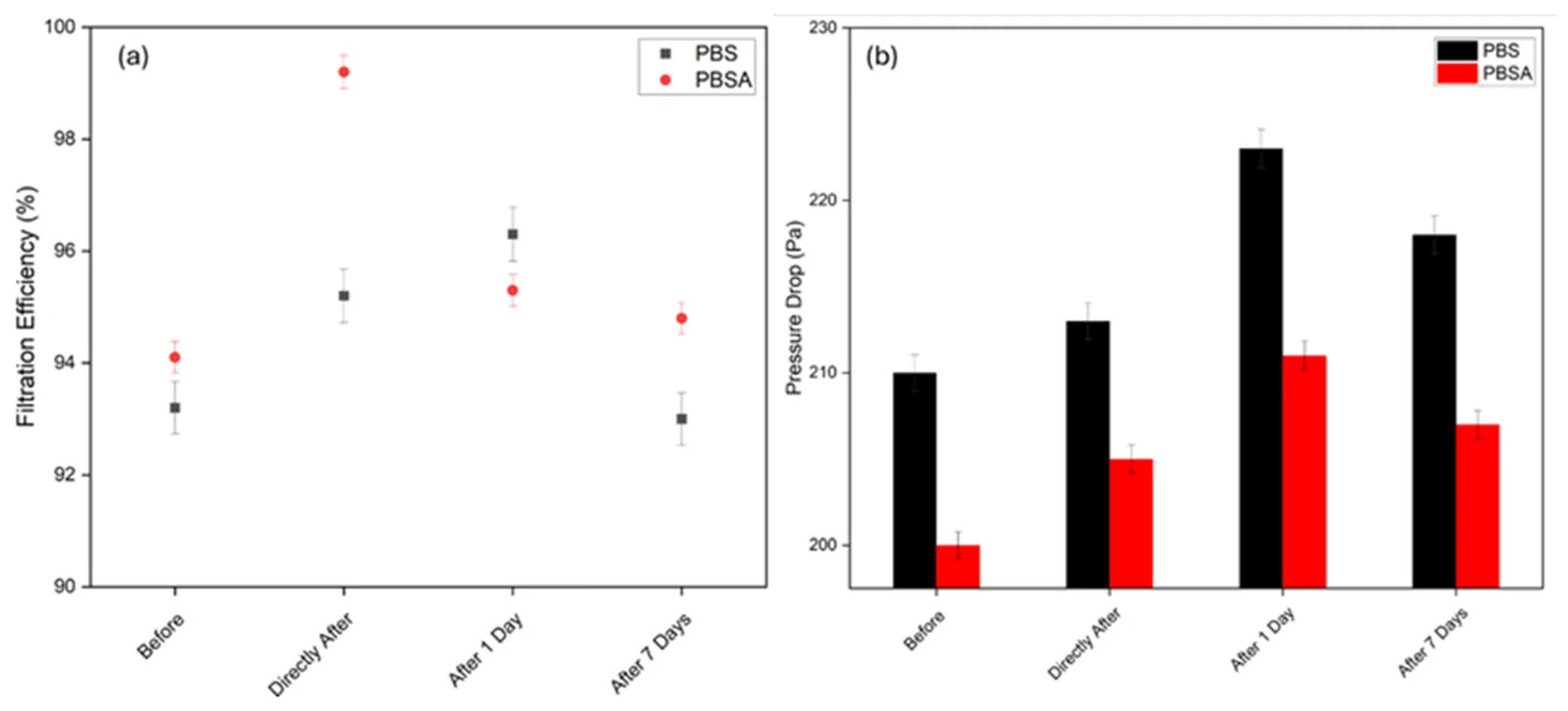
Filtration efficiency and pressure drop of the PBS and PBSA nanofibres before, directly after, after 1 day (**a**) and after 7 days (**b**) of corona discharge.

Figure 8a shows the filtration efficiency of PBS and PBSA nanofibres before corona discharge, immediately after treatment, and after 1 and 7 days of storage. Before corona treatment, both materials exhibited high filtration efficiencies of around 93.2% and 94.1% for PBS and PBSA, respectively. After corona discharge, a clear improvement was observed for both nanofibres, reaching up to 95.2% and 99.2%, respectively. The increase was particularly pronounced for PBSA, which reached its highest efficiency immediately after treatment. After one day, the filtration efficiency of PBS was not affected, while the efficiency of PBSA declined to 95.3% due to the higher hydrophilicity of PBSA, which hindered the stability of electrostatic charges due to moisture accumulation [23]. This reduction became more evident after seven days. However, even after 7 days, the efficiencies remained higher than or comparable to the untreated samples, indicating that the corona-induced enhancement was partially retained over time.

Figure 8b presents the pressure drop values for PBS and PBSA nanofibres under the same conditions. Before corona treatment, PBSA exhibited a slightly lower pressure drop of 200 Pa than PBS (210 Pa). Following corona discharge, the pressure drop was not significantly affected as the electrostatic charges did not affect the airflow resistance through the nanofibres. Generally, PBSA nanofibres exhibited better breathability with lower pressure drops values than the PBS sample. To further understand the filtration behaviour of the fabricated membranes, their structural characteristics, including porosity, basis weight and thickness, were evaluated (Table 3). Both PBS and PBSA nanofibre membranes exhibited similar thicknesses of approximately 150 µm, indicating that the observed differences in filtration performance were not influenced by membrane thickness. However, PBSA showed a slightly higher basis weight (20.2 ± 0.5 g m^−2^) than PBS (19.3 ± 0.9 g m^−2^), suggesting a greater amount of deposited fibres per unit area. In addition, PBSA exhibited a higher porosity (90 ± 4.8%) compared with PBS (85 ± 4.7%), while simultaneously possessing a smaller mean pore size (7.2 ± 0.5 µm versus 8.5 ± 0.7 µm). This combination of high porosity and reduced pore size provides a more favourable microstructure for particle capture, as the interconnected pore network facilitates airflow while increasing the probability of particle interception and diffusion. Although the PBSA fibres were coarser than those of PBS, the slightly higher basis weight and more porous membrane structure likely compensated for the increased fibre diameter by producing a denser fibrous network with smaller effective flow channels. Similar observations have been reported for fibrous filtration media, where filtration performance depends not only on fibre diameter but also on the combined effects of pore structure, porosity and fibre packing density, which together govern particle transport and capture efficiency [24]. Therefore, the improved filtration efficiency of the PBSA membrane can be attributed to the synergistic effects of its membrane architecture, together with the contribution of electrostatic charging, rather than fibre diameter alone.

**Figure 9:**
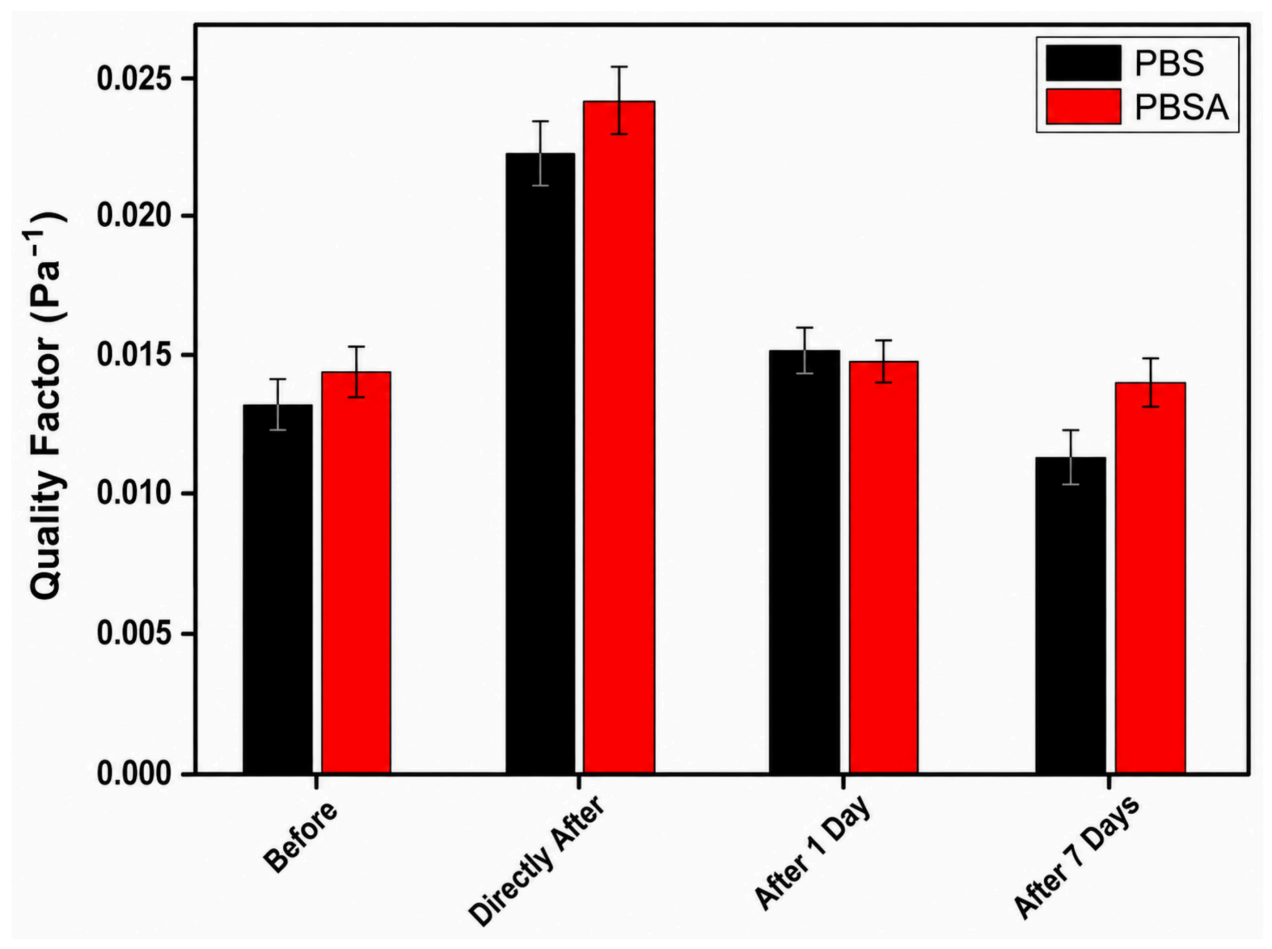
Quality factor of the PBS and PBSA nanofibres before, directly after, after 1 day and after 7 days of corona discharge.

The quality factor (QF), shown in Figure 9, provides a comprehensive evaluation of filtration performance by simultaneously considering filtration efficiency and pressure drop. Since it accounts for both particle removal capability and airflow resistance, it offers a more meaningful comparison of overall filter performance. Before corona discharge, both PBS and PBSA nanofibres exhibited moderate QF values of around 0.0135 Pa-1. Immediately after corona treatment, a significant increase in QF was observed for both materials up to 0.022 Pa-1 for PBS and 0.024 Pa-1 for PBSA. This increase reflected the strong enhancement in filtration efficiency while maintaining a relatively controlled rise in pressure drop. After one day, the QF decreased for both materials, indicating partial loss of electrostatic effects induced by corona discharge.

The reduction continued after seven days, particularly for PBS, which showed the lowest QF at this stage (0.011 Pa-1). In contrast, PBSA maintained comparatively higher QF values even after 7 days, suggesting better retention of electrostatic charge and more stable long-term performance. Corona discharge substantially improved the quality factor of both PBS and PBSA nanofibres. PBSA demonstrates superior overall performance across all time points, achieving a better balance between high filtration efficiency and low pressure drop. These results confirm that PBSA nanofibres exhibit more stable and efficient filtration behaviour following corona treatment.

## 4. Conclusions

This study demonstrates the differences between PBS and PBSA nanofibres manufactured by the electro-blow spinning technique. The study shows the advantages and disadvantages of each biodegradable polymer through detailed morphological, structural and mechanical analyses. We found that the PBS nanofibres have higher crystallinity of 45% while the PBSA nanofibres showed lower crystallinity of 21.5%. The PBS pretended single fibre formations while the PBSA tended to form bundled or merged fibres. Moreover, both the PBS and PBSA showed nearly similar contact angles, while the PBSA had a slightly larger pore size compared to the PBS.

Regarding the mechanical analysis, the PBS had a significantly high stress up to 6 MPa with outstanding young’s modules of 150 MPa. In contrast, the PBSA nanofibres had higher elongation reaching up to 35%, which is almost double that of the PBS. These differences in morphological, chemical and mechanical properties are crucial in determining the suitability of each material for applications, including filtration, wound healing and food packaging, and highlight the importance of PBS and PBSA as common biodegradable polyesters. In terms of filtration performance, PBSA showed enhanced filtration efficiency and pressure drop than the PBS membrane. Both materials showed sustained electrostatic charges for up to 7 days after the corona treatment, which confirms their suitability for long-term air filtration applications.

## Figures and Tables

**Table 1:** Chemical, thermal and mechanical properties of PBS and PBSA biodegradable polymers.

Property	PBS	PBSA
Density (kg/m^3^)	1.26	1.24
Melting Point (°C)	115	84
Yield Stress (MPa)	40	17
Stress at Break (MPa)	36	24
Strain at Break (%)	210	380
Flexural Modulus (MPa)	650	250
Reference	[11]	[11]

**Table 2:** Mechanical properties of PBS and PBSA nanofibres.

Property	PBS	PBSA
Max Strength (MPa)	6 ± 0.5	2 ± 1.8
Max Strain (%)	17 ± 5	35 ± 3
Young’s Modulus (MPa)	150 ± 18	15 ± 10
Toughness (J/m^3^)	172.3 ± 17.2	129.8 ± 13

**Table 3:** Properties of PBS and PBSA nanofibres.

Sample	Mean Diameter (nm)	Mean Pore Size (µm)	Contact Angle (°)	Porosity (%)	Thickness (µm)	Basis Weight (g/m^2^)
PBS	184 ± 32	8.5 ± 0.7	139 ± 2	85 ± 4.7	150 ± 3	19.3 ± 0.9
PBSA	452 ± 24	10 ± 0.5	135 ± 3	90 ± 4.8	150 ± 5	20.2 ± 0.5

## Data Availability

Dataset available on request from the authors.
